# Protein kinase PIM2: A simple PIM family kinase with complex functions in cancer metabolism and therapeutics

**DOI:** 10.7150/jca.53134

**Published:** 2021-03-05

**Authors:** Yixin Wang, Jing Xiu, Chune Ren, Zhenhai Yu

**Affiliations:** Department of Reproductive Medicine, Affiliated Hospital of Weifang Medical University, Weifang, Shandong Province, P.R. China.

**Keywords:** PIM2, oncogene, protein kinase, phosphorylation, inhibitors

## Abstract

PIM2 (proviral integration site for Moloney murine leukemia virus 2) kinase plays an important role as an oncogene in multiple cancers, such as leukemia, liver, lung, myeloma, prostate and breast cancers. PIM2 is largely expressed in both leukemia and solid tumors, and it promotes the transcriptional activation of genes involved in cell survival, cell proliferation, and cell-cycle progression. Many tumorigenic signaling molecules have been identified as substrates for PIM2 kinase, and a variety of inhibitors have been developed for its kinase activity, including SMI-4a, SMI-16a, SGI-1776, JP11646 and DHPCC-9. Here, we summarize the signaling pathways involved in PIM2 kinase regulation and PIM2 mechanisms in various neoplastic diseases. We also discuss the current status and future perspectives for the development of PIM2 kinase inhibitors to combat human cancer, and PIM2 will become a therapeutic target in cancers in the future.

## Introduction

In 1989, PIM2 kinase was found in the proviral insertion site of the Moloney murine leukemia virus. PIM2 is a serine/threonine kinase belonging to the PIM kinases family, along with two other members named PIM1 and PIM3 1, 2, and all three family members belong to the Ca2+/calmodulin-dependent group of protein kinases. The PIM2 protein plays an important role in promoting cell survival and preventing apoptosis. Previous studies have shown that the PIM2 kinase gene is a single-copy gene located on the X chromosome 3. All PIM mRNA transcripts are encoded by six exons and have large 5'- and 3'-UTRs with G/C-rich regions, and five copies of destabilizing AUUUA motifs. PIM1 has two protein isoforms (34 and 44ΚDa), both containing a proline-rich N-terminal motif that binds to the ETΚ-SH3 domain and is recruited to cell membranes. PIM2 has there protein isoforms (34, 37, and 40 KDa), but interestingly, only one protein subtype has been detected for the PIM3 transcript 4. In human cells, the PIM2 kinase has only two isoforms (34 and 41ΚDa), but in mice there are there (34, 37, and 40ΚDa), and the 34KDa isoform has been the main focus of many studies because it plays a crucial role in tumor progression. At the amino-acid level, PIM2 has a shared sequence of 55% with PIM1 and PIM3 3. Thus, it also has a similar carcinogenic function as those of PIM1 and PIM3. Although these three proteins are common in cells, they are expressed at different levels in different tissues. PIM1 protein expression is higher in hematopoietic cells, PIM2 protein expression is higher in the brain and in lymphocytes, and PIM3 protein expression is higher in the kidneys, breast, and brain cells 5.

**Figure 1:**
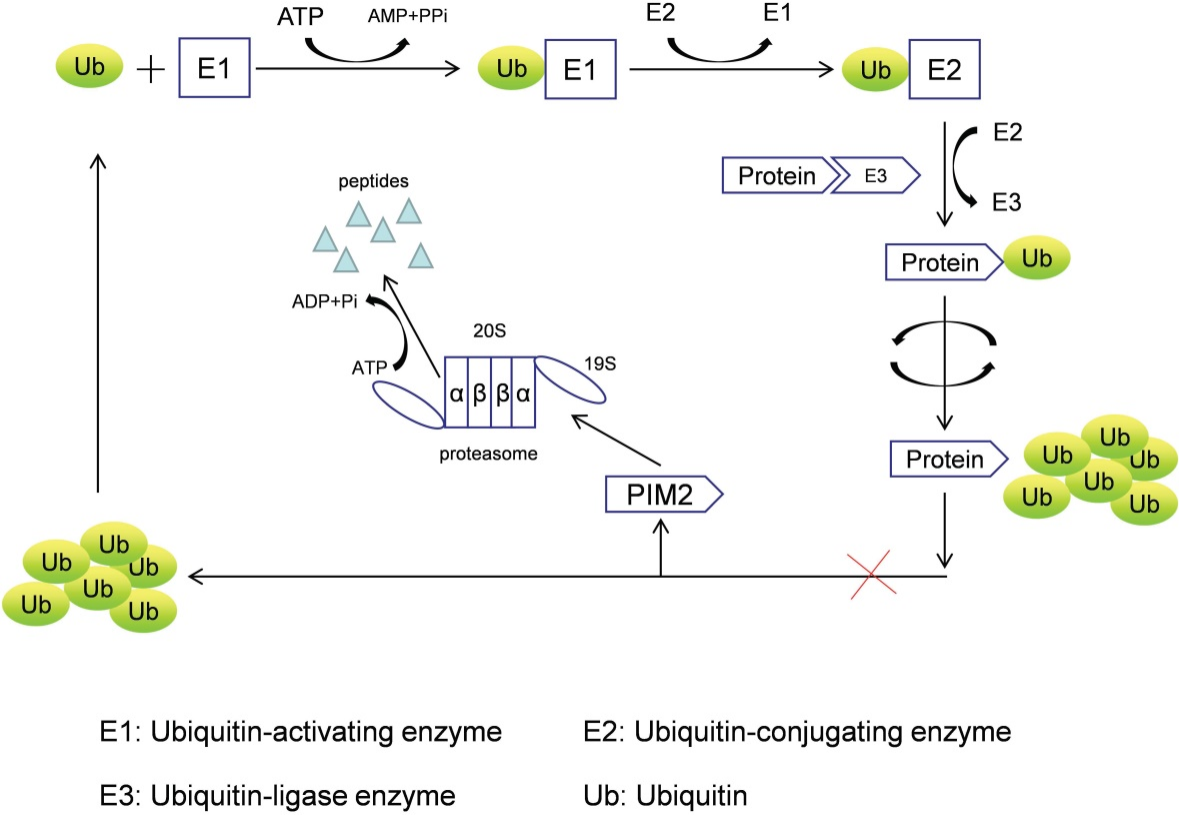
The proteasome degradation pathway of PIM2.

PIM2 is a short-lived protein (<5 min) that does not require any post-translational modifications to induce kinase activation, and it is mainly regulated by protein stability 5, with its degradation achieved by the proteasome pathway. PIM2 aggregation occurs after treatment with proteasome inhibitors (LLnL, bortezomib, lactacystin, and epoxomicin), and PIM2 protein can be rapidly degraded by the proteasome without phosphorylation and dephosphorylation regulatory mechanisms. In addition, many proteins need to be ubiquitinated prior to proteasomal degradation, and the formation of such protein complexes requires cullin scaffold proteins. In general, MLN4924 suppresses these ubiquitinating complexes by inhibiting NEDD8-activating enzyme, but we have found that MLN4942 does not stabilize any of the PIM2 isoforms in UTTAML cells. Therefore, we conclude that PIM2 degradation does not involve the cullin-containing ubiquitin ligase complex, and PIM2 is instead degraded directly by the proteasome pathway (Figure 1) 6.

**Figure 2:**
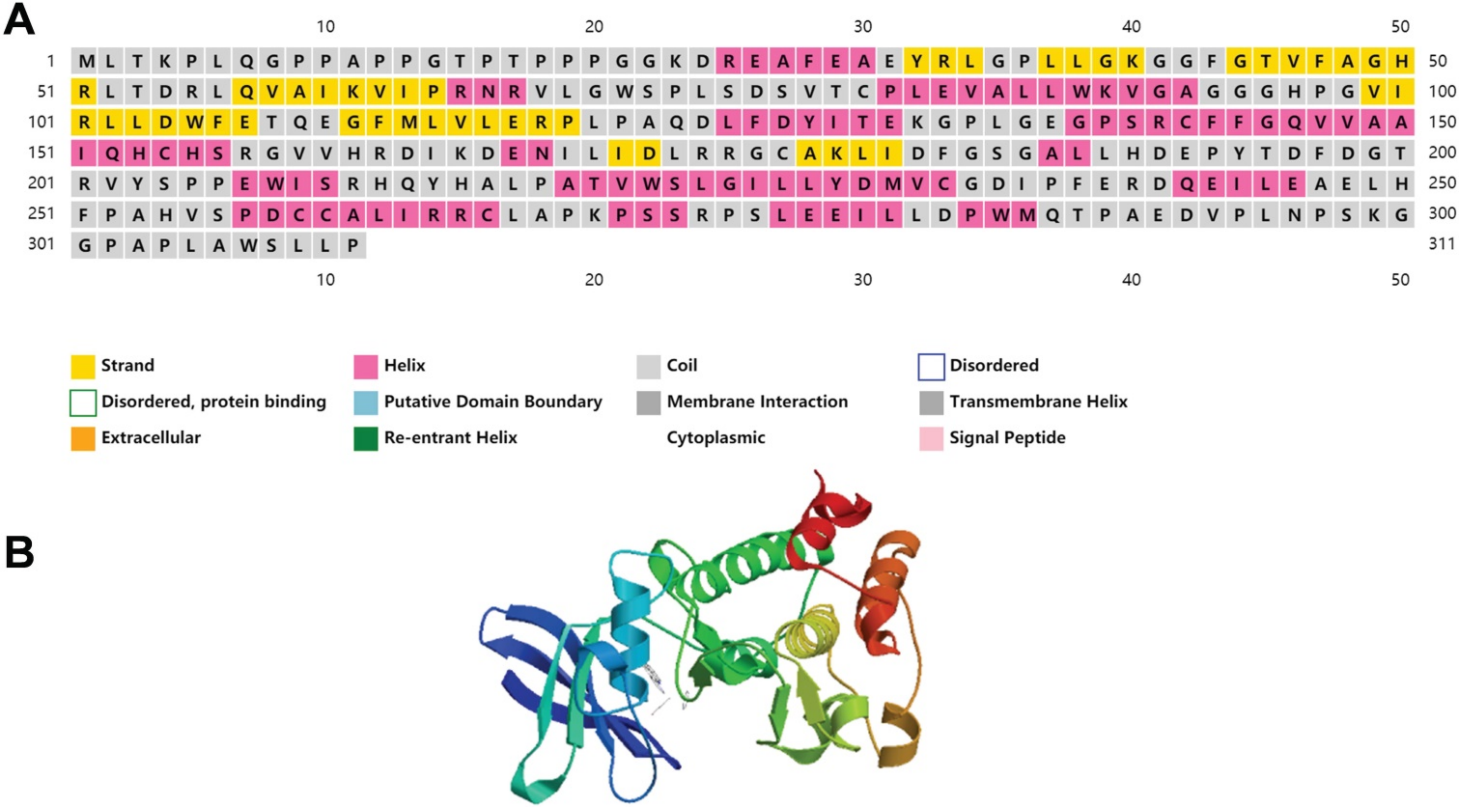
The structure of PIM2 protein kinase.

We have included the secondary and tertiary structures of PIM2 protein (Figure 2), and the three-dimensional structural representations for the three PIM kinases overlapped to show their structural similarities and differences that have been published to have a more intuitive understanding of PIM2 kinase 7. Recent research has demonstrated that PIM2 kinase plays an important role in tumorigenesis, especially in multiple myeloma, lymphoma, acute myeloid leukemia, and in some solid cancers. In fact, elevated PIM2 kinase levels often suggest poor prognoses. However, the underling mechanisms for the regulation of cancer by PIM2 are not yet fully understood. Here, we will focus on research concerning the role of PIM2 in cancer.

## Transcription factors responsible for PIM2 gene activation

**Figure 3:**
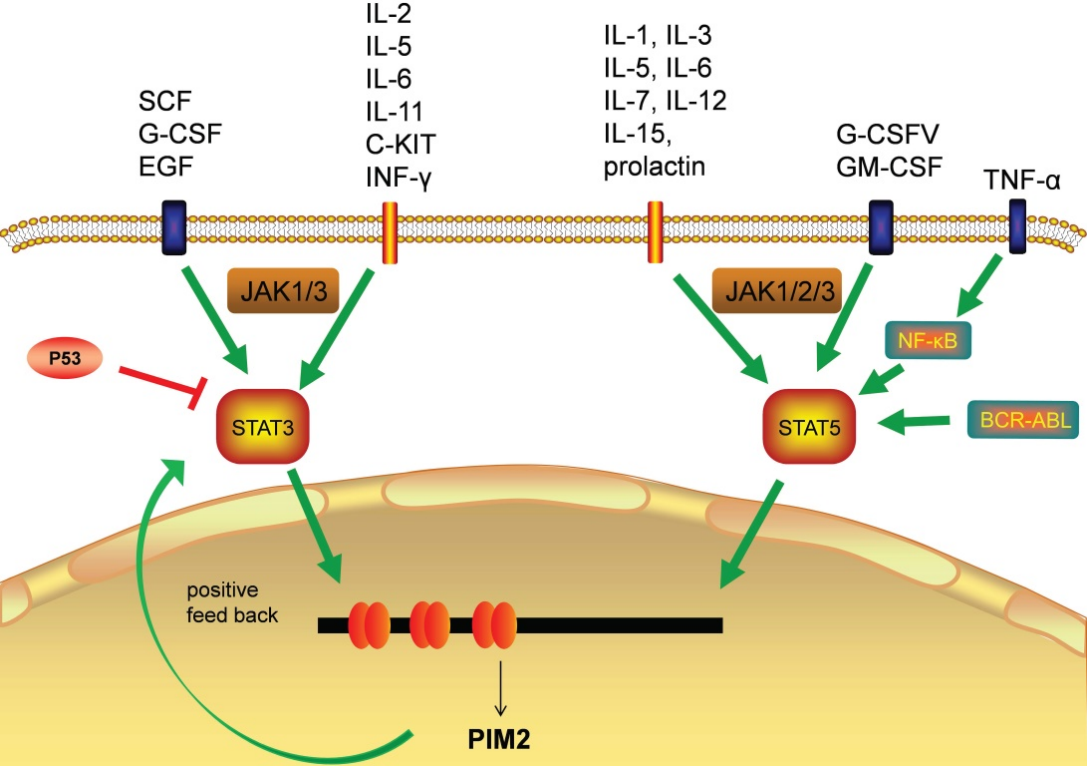
Transcription factors responsible for PIM2 gene transcription.

The transcriptional regulation of PIM2 expression is a complex and sophisticated process that can be influenced by many signaling factors, including the interleukins, GM-CSF, GCSF, and the interferons (Figure 3). Most of these factors have their primary signals transformed through the JAΚ/STAT pathway. Cytokines bind to cell surface receptors that activate the JAΚ/STAT pathway, and the activation of STAT through JAΚ phosphorylation leads to dimerization and nuclear translocation. In the nucleus, these dimers regulate the gene expression by binding to target-specific gene promoter regions: STAT3 and STAT5 bind directly to the PIM2 promoter at the ISFR/GAS-sequence to up-regulate gene expression. Transcription factors downstream of this growth factor signaling pathway, such as NF-κB, can also regulate its expression, as PIM2 has been shown to be up-regulated by NF-κB in response to FLT3/ITB oncogenic mutants 5. The effect of PIM2 on cell survival is dependent on the activation of the NF-κB pathway, which acts as an important mediator. PIM2 activates NF-κB-dependent gene expression by inducing the phosphorylation of serine threonine kinases, leading to an increase in IκB kinase activation, and a shift in nuclear NF-κB from predominantly P50 homodimers to P50/P65 heterodimers. The expression of PIM2 enhances both IκB phosphorylation and degradation, and also promotes NF-κB-dependent cell survival *in vivo*. In addition, the cooperation between PIM2 and MYC depends on the sustained activation of NF-κB 8.

In hepatocellular carcinoma (HCC), the expression of PIM2 has been shown to be both significantly up-regulated and associated with poor patient prognoses. Clinically, the up-regulation of PIM2 has been significantly associated with vascular invasion, recurrence, and tumor node metastasis staging. In HCC, PIM2 expression is highly carcinogenic, and its over-expression may induce the activation of NF-κB signaling pathway through up-regulation of phosphorylated of RIPK2 9. The pathways responsible for STAT3 and STAT5 (normally inactive in the cytoplasm) are first activated by cytokines, and after they enter the nucleus, they initiate PIM2 transcription 5. PIM2 can also promote the expression of STAT3 by inducing cytokines, and this activation of STAT3 causes an increase in PIM2 expression, suggesting a positive feedback loop between PIM2 and STAT3. This activation of STAT3 expression has been shown to be maintained by such a PIM2-dependent positive feedback loop 10. Previous studies have shown that PIM2 expression is also promoted by STAT5 6, and additional studies have demonstrate that the expression of PIM2 is also promoted by a BCR-ABL-dependent STAT5-mediated pathway 11.

The binding of HIF-1α to the PIM2 promoter regulates PIM2 expression via a positive feedback loop, HIF-1α induces PIM2 expression through binding to its promoter, which in turn interacts with HIF-1α to enhance the response to hypoxia 12. In endometrial cancer, PIM2 has been shown to inhibit AMPKα1 kinase activity through direct phosphorylation on Thr-467, leading to the decrease of AMPKα1 kinase activity and the promotion of aerobic glycolysis and tumor growth 13. Tumor cells expressing high level of CHES1 have a low PIM2 expression levels, and CHES1 has been shown to decrease cell proliferation and protein synthesis through its directly binding to the PIM2 promoter 14. Moreover, signaling through DNA double-strand breaks induced by recombination activating genes (RAG) has been shown to induce the expression of PIM2 kinase, which in turn promotes both pre-B cell survival and their G1-S checkpoint 15. The down-regulation of miRNA-135-5p has also been reported to improve the survival rate of skin allografts by up-regulating PIM2 kinase through a negative relationship 16.

## Substrates and the signaling pathways regulated by PIM2

**Figure 4:**
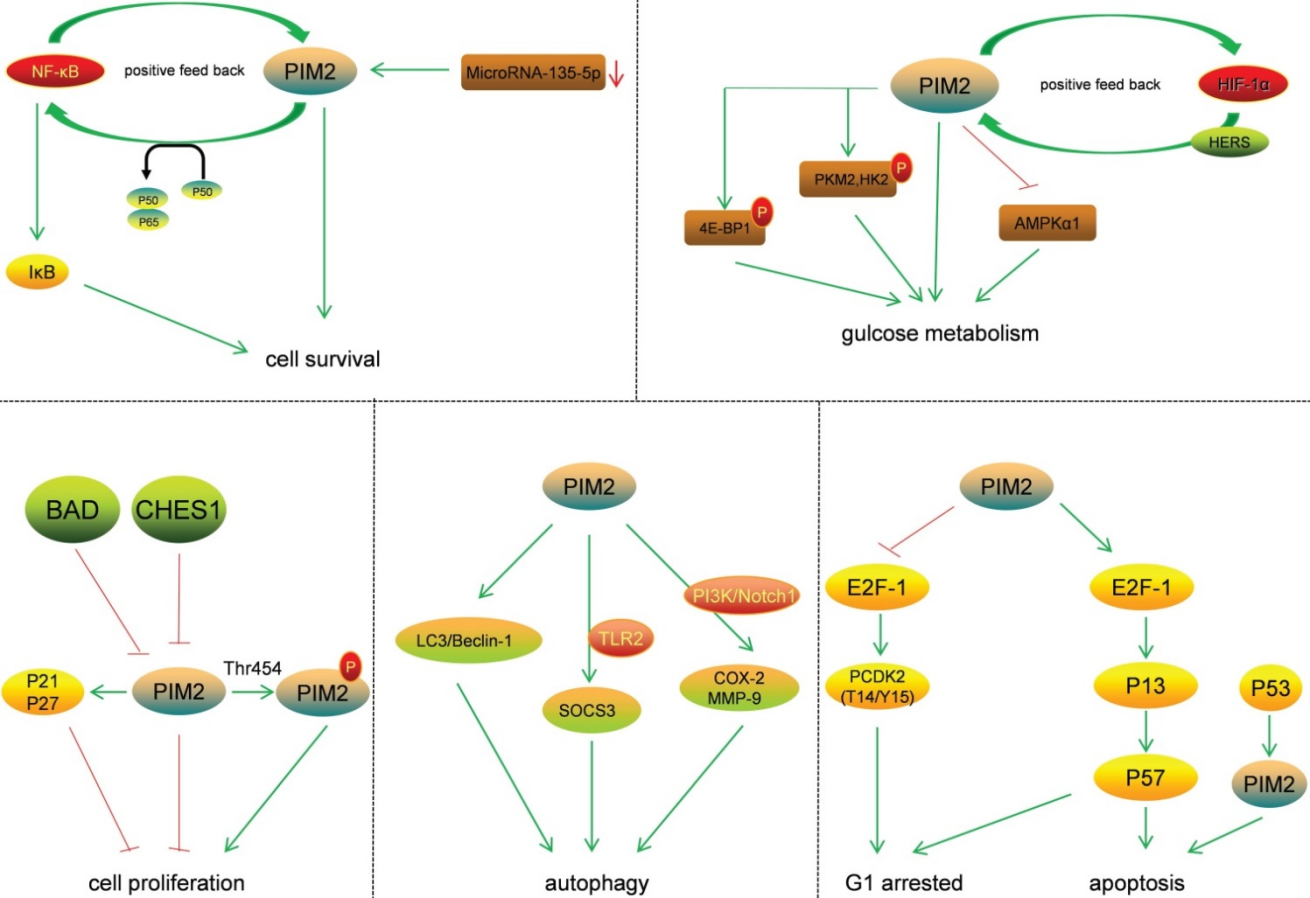
Substrates and the signal pathways regulated by PIM2.

The effect of PIM2 on signaling pathways plays an important role in cancer regulation (Figure 4). The two protein isoforms (34 and 41KDa) in human cells share an identical catalytic site but differ at their N-terminus. As the 34KDa isoform has different nuclear and cytoplasmic functions, the transient expression of the HA-tagged (N-terminus) form of the 34KDa isoform in HeLa cells resulted in an increase in both G1 arrested and apoptotic cells. This was associated with an increase in T14/Y15 phosphorylation of CDΚ2, a proteasome-dependent down-regulation of CDC25A, and up-regulations of p57, E2F-1, and P73 17. The phosphorylation of p21 is known to increase its stability and inhibit cell proliferation in HCT116 cells, and PIM2 kinase is known to phosphorylate p21 both *in vitro* and *in vivo* on Thr-145. Thus, PIM2 may stabilize p21 and increase its stability in the nucleus. In addition, PIM2 kinase has been shown to inhibit cell proliferation through the phosphorylation of p21, and increased p21 expression in the nucleus could potentially lead to cell-cycle arrest 18. The phosphorylation of p27 by PIM2 reduces its stability and promotes its nuclear localization, leading to reduce cell proliferation 19. Moreover, in A549 cells, antibody arrays are used to detect the phosphorylation of BAD on Ser-136, and the results demonstrate that this phosphorylation strongly activates the p53 gene, and that this was accompanied by an accumulation of PIM2 kinase. In contrast, when p53 expression is knocked down, the activation of PIM2 is reduced. In A549 cells, western blot and flow cytometry analyses show that p53 deletion results in reduced camptothecin-induced apoptosis and the proportion of cells with sub-G1 DNA content 20.

PIM2 is highly expressed in asthma patients and primarily located in regulatory T cells (Tregs). The mechanism for this is that PIM2 inhibits IL-10 production by regulating FOXP3, demonstrating that PIM2 is essential for airway inflammation 21. PIM2 is highly expressed and interacts with FOXP3 in human Treg cells. It has been shown to phosphorylate FOXP3 both *in vitro* and *in vivo*, and the elimination of endogenous PIM2 has been reported to enhance the suppressive function of Tregs 22. Not only has PIM2 protein been shown to stabilize c-MYC via phosphorylation at Ser-329, and to enhance its transforming activity in tumorigenesis, but also to modulate endogenous c-MYC levels 23.

PIM2 is known to regulate glucose metabolism in cancer cells, and PIM2-mediated aerobic glycolysis has been shown to be crucial for colorectal tumor cells in concert with mTORC1 signaling 24. Such PIM2-dependent survival is known to be glucose-dependent, and the constitutive expression of PIM2 also confers apoptotic resistance. This anti-apoptosis PIM2 function has been shown not to be dependent on PI3K signal transduction mediators, and endogenous levels of PIM2 contribute to growth factor-induced apoptotic resistance 25. PIM2 mediates glucose metabolism by phosphorylating 4E-BP1, a translation-repressor protein, resulting in translational regulation independent of mTOR 26. Furthermore, PIM2 may also phosphorylate PΚM2 and HΚ2 (key glycolytic enzymes) to promote glycolysis in cancer cells. PIM2 has been shown both to interact with HK2 and phosphorylate it at Thr-473, and to regulate HK2 protein stability via a CMA pathway. Moreover, the phosphorylation of HK2 by PIM2 has been shown to promote glycolysis and autophagy induced by glucose starvation 27. PIM2 has also been found to be a novel binding partner for PKM2, and PIM2 has been shown to phosphorylate PKM2 at Thr-454. This phosphorylation of PKM2 increased cancer cell proliferation, and PIM2 promoted PKM2-dependent glycolysis while reducing mitochondrial respiration 28. Regarding autophagy, PIM2 promoted the expression and organization of the autophagic proteins LC3 and Beclin-1, and led to increases in lysosomal acidification 29. PIM2 has also been reported to induce SOCS3 expression in a TLR2-dependent manner, and SOCS3 is known to be involved in activation of the PIΚ3 and PΚC signaling pathways 30. In macrophages, PIM2 has been shown to induce the expressions of both COX-2 and MMP-9, and the PI3Κ and Notch1 signaling pathways act as early upstream signaling events in this process 31. PIM2 therefore has important roles in cancer via the phosphorylation of essential substrates and the regulation of key signaling pathways.

## PIM2 functions in cancer

Phosphorylation regulates protein activity, stability, subcellular localization, and protein-protein interactions, and is associated with multiple signaling pathways and diseases. As a kinase, the function of PIM2 mainly depends on its ability to phosphorylate, and it plays a large role in cancer (Table 1).

### Leukemia

Both acute lymphoblastic leukemia (ALL) and chronic lymphocytic leukemia (CLL) are hematological malignancies characterized by continuous neoplastic cell trafficking 32. In acute myeloid leukemia (AML) patients, the level of PIM2 kinase is increased, and this high level of PIM2 has been shown to promote tumorigenesis through the phosphorylation of 4E-BP1 26. The same phenomenon is also observed in CLL patients, where PIM2 expression has been reported to be significantly higher in their leukemic cells compared to its expression in peripheral blood lymphocytes of controls. Research has also shown that PIM2 and NF-κB gene expressions are both increased in ALL and CLL patients 33. This elevated expression of PIM2 is associated with poor prognoses in ALL patients, resulting from an increased resistance in leukemic cells to apoptosis, and its clinical significance is associated with low complete-remission rates, shorter leukemia-free survival times, higher-risk cytogenetics, and shorter event-free survival times 34. In CLL patients, PIM2 expression was also higher than in controls, and this was associated with a more rapid lymphocyte-doubling time, and the proportion of malignant lymphocytes 35. PIM2 has been shown to interact with PML-RARα (promyelocytic leukemia/retinoic acid receptor alpha), an association which may induce AML-like diseases, as transplanted PIM2-PML-RARα bone marrow cells induced an AML-like disease in mice, and their spleen cells expressed both PIM2 and PML-RARα 36. Recent studies have shown that in myelodysplastic syndromes (MDS), AML patients had high expression of PIM2 in CD34^+^ cells derived from bone marrow. Such high PIM2 expression is thought to induce HIF1α expression by reducing the expression of IDH1, resulting in the proliferation of CD34^+^ cells 37. It is well known that chronic myeloid leukemia stem cells (CMLSCs) expressing PIM2 increase the resistance to the drug imatinib mesylate (IM). PIM2 expression in CMLSCs is promoted by a BCR-ABL-dependent STAT5-mediated pathway, and as PIM2 phosphorylates and inhibits the pro-apoptotic protein BAD, this maintenance of BAD phosphorylation causes IM resistance 11. In AML, PIM2 mainly controls apoptosis through BAX expression and mitochondrial disruption. Recent research has identified RSΚ2 (encoded by the RPS6KA3 gene) as a new PIM2 targets, and its ectopic expression rescued the viability of PIM2-depleted cells, suggesting that RSΚ2 is downstream of PIM2 in pathways regulating AML cell survival 38.

### Multiple myeloma

Multiple myeloma (MM), characterized by an abnormal increase in monoclonal paraprotein and the accumulation of cancerous plasma cells, is a progressive and debilitating malignancy 39. Research has shown that PIM2 kinase is overexpressed in MM cells, and that the kinase inhibitors SMI-16a and SMI-4a can both reduce its kinase activity in MM cells 40. PIM2 expression has also been shown to be up-regulated in bone marrow stromal cells, acting as a negative regulator for osteoblastogenesis, and PIM2 inhibition suppressed MM tumor progression and prevented bone destruction *in vivo* 41. PIM2 has also been shown to be involved in repressing the DNA-damage response (DDR) by repressing the activation of the DDR pathway via ATR modulation, and knockdown of PIM2 resulted in the up-regulation of downstream DDR markers in MM cells 42. In addition, PIM2 has been reported to directly phosphorylate TSC2 at Ser-1798, and to promote TSC2 suppression of mTOR-C1, suggesting a novel PIM2-TSC2-mTOR-C1 pathway driving MM proliferation 43.

### Breast cancer

Breast cancer continues to be a threat to women's health worldwide 44. Recent studies have shown that PIM2 kinase phosphorylates the key glycolytic enzyme HK2 at Thr-473 and regulates its protein stability in breast cancer cells 27. Over-expression of PIM2 kinase reduced the protein levels of tristetraprolin (TTP) and led to the progression of breast cancer. PIM2 has been shown to bind to the tandem zinc finger domain of TTP in human breast cancer, and the expression of PIM2 and TTP have been reported to be negatively correlated 45. For epithelial-mesenchymal transformation (EMT) in breast cancer, STAT3 (a critical signaling node in EMT) may be part of a positive feedback loop with PIM2 kinase that contributes to the progression of breast cancer 10. In addition, PIM2 physically interacts with HSF1 and phosphorylates it at Thr-120. Indeed, PIM2 may promote HSF1 protein stability via the ubiquitin proteasome pathway because the phosphorylation of HSF1 on Thr-120 has been shown to regulate both proteostasis and carboplatin induced-autophagy that promote breast tumor growth. PIM2-mediated phosphorylation of HSF1 at Thr-120 also induces HSF1 binding to the PD-L1 promoter and enhances PD-L1 expression, which also promotes tumor growth in breast cancer 46.

### Hepatocellular carcinoma

Hepatocellular carcinoma (HCC) is the third leading cause of cancer death worldwide 47. In HCC tissues, miR-26b-5p has been shown to negatively regulate PIM2 kinase, and a knockdown of PIM2 reversed the immunosuppression mediated by anti-miR-26b-5p. Treatment with miR-26b-5p enhanced T-cell responses by targeting PIM2 in HCC, and the absence of PIM2 also enhanced the miR-26b-5P-mediated T-cell response 48. PIM2 mRNA and protein levels have also been reported to be significantly higher in HCC tissues, and the mRNA levels and activities of NF-κB parallel those of PIM2. Through the NF-κB pathway, PIM2 has been shown to activate API-5 and to inhibit apoptosis in HCC cells 49.

### Liver cancer

PIM2 has been shown to promote liver tumorigenesis through mediating cell survival and preventing liver cell apoptosis 50. In the L02 human liver cell line, the stable ectopic expression of the PIM2 gene induces malignant transformations 51, and knockdown of PIM2 in liver cancer results in potent anti-proliferative effects on cell growth G0/G1 cell-cycle blockade and the down-regulation of S phase and G2/M phase genes 52.

### Reproductive system tumor

PIM2 has been shown to be overexpressed in human ovarian and uterine tumors, and this over-expression provokes both tissue alterations and a large IL-6 dependent inflammatory response 53. PIM2 is also induced by cisplatin in ovarian cancer cells, and the targeting of the PIM2 kinase by biochemical inhibitors or RNA interference leads to the reduction of cell growth, the decrease of BAD phosphorylation, and the sensitization of the ovarian cancer cells to drug-induced apoptosis 54. PIM2 is overexpressed in male mice reproductive system in transgenic mice, which regulates the proliferation of male sex organs in mice. The over-expression of PIM2 kinase has also been observed in prostate tumors and in human male germ cells and has been correlated with both inflammatory features and stem-cell markers 55. XIAP, a downstream factor in the PIM2 pathway in prostate cancer, has been shown to cooperate with PIM2 to inhibit apoptosis in prostate cancer cells 56. In endometrial cancer, the phosphorylation of AMPΚα1 on Thr-467 by PIM2 results in less AMPΚα1 kinase activity, which in turn promotes tumor growth 13.

### Other cancers

PIM2 has been shown to promote stomach cancer progression by regulating apoptosis during reactive oxygen species-triggered endoplasmic reticulum stress. It have previously reported up-regulated PIM2 in human gastric cancer specimens, along with increased gastric cancer cell invasion and migration. Furthermore, *in vivo* experiments have revealed that the knockdown of PIM2 inhibits the growth of gastric tumors 57. PIM2 is known to be highly expressed in lung cancer, and using a microarray analysis to detect genes associated with cell proliferation 58. Further, PKM2 translocates to the nucleus after Thr-454 phosphorylation by PIM2, which decreases mitochondrion function and enhances the pentose-phosphate pathway, and ultimately enhances both cell proliferation and tumor growth 59. PIM2 kinase therefore plays a key role as an oncogene in multiple cancers. However, the functions of PIM2 kinase protein in tumorigenesis have not yet been fully elucidated, and additional research is needed to explore its functions in cancer.

## The value of treatments with PIM2 inhibitor

The PIM2 protein has been shown to be involved in tumorigenesis, promoting tumor growth, and inducing chemotherapy resistance. In addition, high levels of PIM2 kinase have been associated with poor prognoses in tumor patients, suggesting that its inhibition may be a promising new therapeutic strategy against cancer. In addition, PIM2 has been identified as a clinical biomarker and a potential therapeutic target for the individualized treatment of cancers 60, 61. Not only has the involvement of PIM2 kinase created broad interest in oncology research due to its over-expression in cancer, but the use of PIM2 inhibitors may also identify other signaling pathways involved 62. The crystal structure of PIM2 complexed with organoruthenium (inhibition at the sub-nanomolar level) may also be used to develop highly potent inhibitors for the PIM kinases 3. Here, we summarize PIM2 inhibitors and their effects on a variety of tumors (Table 2).

### SMI-4a

SMI-4a has been reported to be a PIM2-specific kinase inhibitor that can abrogate the effects of phosphorylated HΚ2 (Thr-473) on paclitaxel resistance in breast cancer. To verify this, we conducted a double assay using both MCF-7/TaxR cells and xenografts in nude mice, and as expected, the combination of paclitaxel and SMI-4a proved most efficient for inhibiting MCF-7/TaxR cell growth 27. In both leukemia and lymphoma, SMI-4a has been shown to block the growth of precursor T-cells by inhibiting PIM2, and SMI-4a treatment of leukemic cells induced cell-cycle arrest by inducting apoptosis. Regarding cellular pathways, SMI-4a has been shown to inhibit the mTOR pathway to up-regulate the MAPK pathway, and ultimately to reduce leukemic cell growth *in vivo* 63. In B-ALL patients, PIM2 has been reported to be overexpressed in combination with high HO-1 levels, and treatment with SMI-4a decreases this HO-1 expression and inhibits B-ALL cell viability. In addition, by down-regulating BCL-2, SMI-4a also induces cellular apoptosis. Overall, through the HO-1-mediated JAΚ2/STAT3 pathway, SMI-4a inhibits B-ALL proliferation and induces apoptosis 64. In melanoma, SMI-4a inhibits tumor growth by inducing autophagy through the down-regulation of AΚT/mTOR axis 65. Moreover, SMI-4a treatment of T-LBL inhibits the mTOR pathway and up-regulates the MAPΚ pathway, which decreases the growth of leukemic cells *in vivo* 63. In hematopoietic and prostate cells, SMI-4a causes a dose-dependent reduction in p-BAD, and administration of SMI-4a to PC3, LNCaP, DU745, K562, and U937 cells results in different degrees of inhibition (10-40%) after 72hr of treatment.

### AZD1208

In 93T449 human liposarcoma cells, AZD1208 strongly inhibits cell growth, and reduces STAT3 phosphorylation and cell survival. By inhibiting mTOR, S6K and 4E-BP1, AZD1208 also inhibits the growth of AML cells. In addition, AZD1208 also strongly induces the phosphorylation of AMPK in 93T449 cells via a decrease in cellular ATP rather than through LKB1 66. In hepatoblastoma, PIM2 has been shown to promote tumorigenesis. By inhibiting PIM2, AZD1208 decreases the proliferation, migration, and invasiveness of long-term passaged hepatoblastoma cells and increases apoptosis. In addition, tumor growth in mice is decreased after AZD1208 treatment, with approximately 57% tumor regression 67. Treatment with AZD1208 has also been shown to suppress the Ser-112 phosphorylation of proapoptotic BAD. In both AML cell lines and in xenograft tumor models, AZD1208 inhibits the growth in the cells and tumors. These results suggest that inhibiting PIM2 with AZD1208 may be beneficial for the treatment of FLT3 wild-type and FLT-ITD AML. CLL cells have been reported to have higher levels of both PIM2 protein and mRNA compared to normal lymphocytes, and treatment of CLL lymphocytes with AZD1208 results in toxic cell death, but no such cytotoxicity is detected in healthy lymphocytes. Such AZD1208 cytotoxicity after treatment occurs due to the inhibition of translation and the induction autophagy, which causes cell-cycle arrest and cell death 68, 69.

### SMI-16a

Similar to SMI-4a, SMI-16a also suppresses the drug-efflux function of breast cancer resistance protein, and reduces the capacities for both colony formation and tumorigenic activity in MM cells. SMI-16a has been shown to destroy clone formation in MM cells, and their tumorigenic ability *in vivo* under acidic conditions, which restores the anti-MM effects of Dox. The accumulation of PIM2 in MM cells can be reduced using proteasome inhibitors, and SMI-16a has been shown to enhance the cytotoxic effects of carfilzomib by inhibiting the accumulation of PIM2 by proteasome inhibitors 40. PIM2 has also been shown to act as a negative regulator for osteoblastogenesis. The inhibition of PIM2 can affect the formation of osteoblasts, and the expression of PIM2 can be up-regulated by the effect of inhibitory factors on the formation of MM osteoblasts.* In vivo*, PIM2 inhibition blocks the progression of MM tumor cells and prevents bone damage. Moreover, reducing PIM2 kinase activity increases and cooperatively enhances anti-MM effects 41.

### JP11646

It is well known that PIM2 is overexpressed in MM, and JP11646 (a selective PIM2 inhibitor) has been shown to inhibit downstream PIM2 molecular targets, such as 4EBP1, BAD, and MCL1. PIM2 inhibition by JP11646 also causes greater inhibition of proliferation and cell viability. In myeloma cells, JP11646 treatment inhibits PIM2 expression, and this inhibition overcomes any compensatory growth factor and pro-survival signaling. JP11646 has been reported to down-regulate both PIM2 mRNA and protein levels, leading to the inhibition of PIM2 kinase activity. Treatment with JP11646 in murine xenogeneic myeloma models leads to significant reductions in tumor burden and the increase of median survival 70.

### SGI-1776

SGI-1776, a pan-PIM inhibitor, has been shown to induce G1 arrest and apoptosis in prostate cancer cells, and to decrease the phosphorylation of p21/waf1 and the BAD pathway 5. SGI-1776 treatment may represent a new strategy against AML because it has been shown to be effective in AML cell lines for inhibiting PIM2 kinase pathways and inducing apoptosis 71. Moreover, the use of SGI-1776 as a drug has been shown to reduce tumor growth in human urothelial carcinomas 72. In both SACC-83 and SACC-LM cells, SGI-1776 has been reported to cause cell-cycle arrest, to reduce cellular proliferation, and to inhibit both cell migration and invasiveness. During the early phases of differentiation, SGI-1776 has also been shown to inhibit adipogenesis. The phase I clinical trial on SGI-1776, which begins in 2008, is halted due to cardiotoxicity caused by the compound 73.

### DHPCC-9

Treatment of prostate cells with DHPCC-9 inhibits the phosphorylation of BAD by PIM kinase and leads to an inhibition of cellular invasion and migration 74. DHPCC-9 has not only been used as a powerful tool to investigate the physiological effects of the oncogenic PIM family of kinases, but it also represents an attractive molecule for drug development to inhibit the invasiveness of PIM-overexpressing cancer cells. DHPCC-9 inhibits the phosphorylation of intracellular PIM substrates, such as BAD. Silencing PIM gene expression reduces cell activity and alleviates migration and metastasis of cancer cells in patients with prostate or squamous cell carcinoma. But it does not affect the cell ability of metabolize and survive 75.

### Other inhibitors

CX-6258 (also known as Compound 13) is also a pan-PIM kinase inhibitor with excellent biochemical potency and kinase selectivity. It has exhibited synergistic activity *in vitro* with chemotherapeutics and robust *in vivo* efficacy in two PIM kinase driven tumor models 76. Few other inhibitors of PIM2 kinase have been reported, indicating that further development of PIM2 inhibitors is needed to treat cancers. Imidazopyridazine has been shown to inhibit the phosphorylation of BAD, resulting in decreased cell proliferation in MM cells via inhibition of PIM kinase activity 77.

## Conclusions

PIM2 kinase research regarding the treatment of tumors has become more intense in recent years. Its functions indicate important roles for it in the processes of cell growth and development, and it may become a key target for cancer treatment. PIM2 acts as a proto-oncogene during the development of a large variety of human tumors. In this review, we have outlined the many possible PIM2 pathways, and each of these may be a potential therapeutic target for treating cancer. Research has demonstrated that PIM2 kinase enhances tumor-cell proliferation both *in vivo* and *in vitro,* and additional inhibitors blocking PIM2 kinase activity have also been developed, providing more strategies to fight cancer. However, the roles of PIM2 vary in different tumors, so the development of useful PIM2-targeting drugs will take perseverance. These crucial roles for PIM2 kinase both in tumor development and transformation indicate that the future targeting of PIM2 kinase may offer better anti-cancer therapeutic regimens.

## Figures and Tables

**Table 1:** Functions of PIM2 in cancer

Cancer	Function	References
Leukemia	1. In AML patients, the level of PIM2 kinase is increased, and this high level of PIM2 promotes tumorigenesis through the phosphorylation of 4E-BP1.	26
	2. The elevated PIM2 expression is associated with poor prognoses in ALL patients, resulting from an increased resistance in leukemic cells to apoptosis.	34
	3. In CLL patients, the high expression of PIM2 was associated with a more rapid lymphocyte-doubling time, and the proportion of malignant lymphocytes.	35
	4. In MDS, patients had high expression of PIM2 in CD34+ cells derived from bone marrow. Such high PIM2 expression is thought to induce HIF1α expression by reducing the expression of IDH1, resulting in the proliferation of CD34+ cells.	37
	5. In CMLSCS, PIM2 expression is promoted by a BCR-ABL-dependent STAT5-mediated pathway and as PIM2 phosphorylates and inhibits the pro-apoptotic protein BAD, this maintenance of BAD phosphorylation causes IM resistance.	11
Multiple myeloma	1. PIM2 has been shown to be involved in repressing the DNA-damage response (DDR) by repressing the activation of the DDR pathway via ATR modulation and knockdown of PIM2 resulted in the up-regulation of downstream DDR markers in MM cells.	42
	PIM2 has been reported to directly phosphorylate TSC2 at Ser-1798, and to promote TSC2 suppression of mTOR-C1, suggesting a novel PIM2-TSC2-mTOR-C1 pathway driving MM proliferation.	43
Breast cancer	PIM2 kinase phosphorylates the key glycolytic enzyme HK2 at Thr-473 and regulates its protein stability in breast cancer cells.	27
	For epithelial-mesenchymal transformation (EMT) in breast cancer, STAT3 (a critical signaling node in EMT) may be part of a positive feedback loop with PIM2 kinase that contributes to the progression of breast cancer.	10
	PIM2-mediated phosphorylation of HSF1 at Thr-120 induces HSF1 binding to the PD-L1 promoter and enhances PD-L1 expression, which also promote tumor growth in breast cancer.	46
Hepatocellular carcinoma	1. In HCC tissues, miR-26b-5p has been shown to negatively regulate PIM2 kinase, and a knockdown of PIM2 reversed the immunosuppression mediated by anti-miR-26b-5p.	48
	2. Through the NF-κB pathway, PIM2 has been shown to activate API-5 and to inhibit apoptosis in HCC cells.	49
Liver cancer	1. Knockdown of PIM2 in liver cancer result in potent anti-proliferative effects on cell growth G0/G1 cell-cycle blockade and the down-regulation of S phase and G2/M phase genes.	52
Ovarian and uterine tumors	PIM2 overexpression provoked both tissue alterations and a large IL-6 dependent inflammatory response.	53
	2. PIM2 was induced by cisplatin in ovarian cancer cells, and the targeting of the PIM2 kinase by biochemical inhibitors or RNA interference reduced cell growth, decreased BAD phosphorylation, and sensitized the ovarian cancer cells to drug-induced apoptosis.	54
Prostate tumors	1. High level expression of PIM2 kinase has been correlated with both inflammatory features and stem-cell markers.	55
	2. XIAP, a downstream factor in the PIM2 pathway in prostate cancer, has been shown to cooperate with PIM2 to inhibit apoptosis in prostate cancer cells.	56
Endometrial cancer	1. The phosphorylation of AMPΚα1 on Thr-467 by PIM2 results in less AMPΚα1 kinase activity, which in turn promotes tumor growth.	13
Stomach cancer	1. Up-regulated PIM2 in human gastric cancer specimens, along with increased gastric cancer cell invasion and migration. And *in vivo* experiments that the knockdown of PIM2 inhibits the growth of gastric tumors.	57
Lung cancer	PIM2 translocates to the nucleus after Thr-454 phosphorylation, and this phosphorylation decreases mitochondrion function and enhances the pentose-phosphate pathway, which ultimately enhances both cell proliferation and tumor growth.	59

**Table 2:** PIM2 inhibitors and their function

Inhibitors	Function	References
SMI-4a	1. Abrogate the effects of phosphorylated HΚ2 (Thr-473) on paclitaxel resistance in breast cancer.	27
	2. Block the growth of precursor T-cells by inhibiting PIM2, and SMI-4a treatment of leukemic cells induced cell-cycle arrest by inducting apoptosis.	63
	3. Through the HO-1-mediated JAΚ2/STAT3 pathway, SMI-4a inhibits B-ALL proliferation and induces apoptosis.	64
	4. Inhibit the mTOR pathway to up-regulate the MAPK pathway, and ultimately to reduce leukemic cell growth *in vivo*.	63
	5. Inhibits tumor growth by inducing autophagy through the down-regulation of AΚT/mTOR axis.	65
AZD1208	1. In 93T449 human liposarcoma cells, AZD1208 strongly inhibits cell growth, reduces STAT3 phosphorylation, and reduces cell survival.	66
	2. By inhibiting mTOR, S6K, S6, and 4E-BP1, AZD1208 inhibits the growth of AML cells.	66
	3. Decreases the proliferation, migration, and invasiveness of long-term passaged hepatoblastoma cells and increases apoptosis.	67
	4. Treatment with AZD1208 has been shown to suppress the Ser-112 phosphorylation of proapoptotic BAD.	68
SMI-16a	1. Suppresses the drug-efflux function of breast cancer resistance protein.	40
	2. Reduces the capacities for both colony formation and tumorigenic activity in MM cells.	40
	3. Destroy clone formation in MM cells, their tumorigenic ability *in vivo* and under an acidic conditions, and to restore the anti-MM effects of Dox.	40
	4. Enhance the cytotoxic effects of carfilzomib by inhibiting the accumulation of PIM2 by proteasome inhibitors.	40
JP11646	1. Inhibit downstream PIM2 molecular targets, such as 4EBP1, BAD, and MCL1. PIM2 inhibitition by JP11646 also causes greater inhibition of proliferation and cell viability.	70
	2. Down-regulate both PIM2 mRNA and protein levels, leading to the inhibition of PIM2 kinase activity.	70
	3. Treatment withJP11646 in murine xenogeneic myeloma models has resulted in significant reductions in tumor burden and increased median survival.	70
SGI-1776	1. Induce G1 arrest and apoptosis in prostate cancer cells, and to decrease the phosphorylation of p21/waf1 and the BAD pathway.	5
	2. SGI-1776 treatment may represent a new strategy against AML because it has been shown to be effective in AML cell lines for inhibiting PIM2 kinase pathways and inducing apoptosis.	71
	3. SGI-1776 as a drug has been shown to reduce tumor growth in human urothelial carcinomas.	72
DHPCC-9	1. Inhibits the phosphorylation of BAD by PIM kinase and leads to an inhibition of cellular invasion and migration.	74
	2. Not only been used as a powerful tool to investigate the physiological effects of the oncogenic PIM family of kinases, but it also represents an attractive molecule for drug development to inhibit the invasiveness of PIM-overexpressing cancer cells.	75
CX-6258	1. Exhibited synergistic activity *in vitro* with chemotherapeutics and robust *in vivo* efficacy in two PIM kinase driven tumor models.	76
Imidazopyridazine	1. Inhibit the phosphorylation of BAD, resulting in decreased cell proliferation in MM cells via inhibition of PIM kinase activity.	77
